# Airway smooth muscle as an underutilised biomarker: a case report

**DOI:** 10.1186/s12890-019-0789-7

**Published:** 2019-01-28

**Authors:** Joy Sha, Steuart Rorke, David Langton

**Affiliations:** 1 Department of Thoracic Medicine, Frankston Hospital, 2 Hastings Road, Frankston, Victoria Australia; 2 Dorevitch Pathology, Heidelberg, Melbourne, Victoria Australia; 3 Faculty of Medicine, Nursing and Health Sciences, Monash University, Clayton, Melbourne, Victoria Australia

**Keywords:** Airway smooth muscle, Asthma, Bronchoscopy, Chronic obstructive pulmonary disease, Endobronchial biopsy

## Abstract

**Background:**

Severe asthma and chronic obstructive pulmonary disease (COPD) can be challenging to manage, particularly when the clinical features may be similar. With the increased availability of advanced therapies for both entities, it is more important than ever to diagnose and phenotype accurately to inform appropriate treatment decisions. This case highlights the use of endobronchial biopsies to allow for histological evaluation of airways disease, and in particular the role of airway smooth muscle mass as an additional biomarker that could facilitate the diagnostic process.

**Case presentation:**

A 65 year old woman presented with a diagnosis of severe COPD on the background of previous smoking and mild childhood asthma. Despite taking maximal inhaled pharmacotherapy, she had frequent exacerbations requiring corticosteroids and remained dyspnoeic on mild exertion. Lung function tests showed severe obstruction on spirometry (forced expiratory ratio 43%, forced expiratory volume in 1 s 47% predicted), and single breath Diffusing Capacity for Carbon Monoxide was moderately reduced at 45% predicted. Computed tomography revealed hyperinflation without marked emphysema. Quantitative CT for emphysema distribution demonstrated a relatively small lung fraction of 9.35% with <− 950 Hounsfield units. Bronchoscopy with endobronchial biopsy was undertaken to further determine the underlying pathology, and airway mucosa histology was consistent with typical findings of asthma. The patient was treated with bronchial thermoplasty as she did not meet prescribing criteria for monoclonal antibodies. Six months post treatment, she had a significant improvement in symptom control and medication usage, without any exacerbations.

**Conclusions:**

Airway smooth muscle histology is an underutilised biomarker that has a valuable role in phenotyping airways disease in the era of individualised medicine.

## Background

There is well-recognised overlap between asthma and chronic obstructive pulmonary disease (COPD) manifestations. The diagnostic uncertainty can increase at the severe end of the spectrum of both diseases when symptoms are difficult to control despite maximal conventional therapy. It is in these severe cases that advanced therapies have become available, including monoclonal antibodies and bronchial thermoplasty (BT) for asthma, and endobronchial valves for COPD. Arriving to the correct diagnosis and disease phenotype is of utmost importance for treatment eligibility and patient care. This case describes a patient whose diagnosis and course of treatment became significantly altered following results of endobronchial biopsies.

## Case presentation

A 65 year old woman was referred for a second respiratory opinion for persistent MRC grade 4 dyspnoea on a background of chronic obstructive pulmonary disease (COPD). Relevant past history included 33 pack years smoking history with smoking cessation 12 months prior, childhood history of mild asthma, and mild diastolic dysfunction. She required two hospital admissions for exacerbations in the past 12 months, in addition to multiple courses of oral corticosteroids. She had been adherent to her medications, which included total daily doses of budesonide/eformoterol 800/24mcg, ciclesonide 320mcg, aclidinium 322mcg, theophylline slow release 600 mg, and doxycycline 50 mg. In addition, she nebulised salbutamol 5 mg each morning, and took 6-8 additional puffs of salbutamol during the day.

On examination, her body mass index was 25, with normal vital signs and oxygen saturation 95% breathing room air. There was no finger clubbing. The chest was hyperinflated and there were no adventitial sounds.

Spirometry revealed severe airflow obstruction with a forced expiratory ratio of 43% and forced expiratory volume in 1 s (FEV_1_) of 47% predicted (0.86 L), with a partial bronchodilator response (130 ml and 15.6%). Gas trapping was evident with an elevated residual volume (RV) of 189% predicted, and a Residual Volume to Total Lung Capacity ratio of 55%. The single breath Diffusing Capacity for Carbon Monoxide was measured at 8.2 ml/mmHg/l or 45% predicted. Arterial blood gases were not measured as SpO2 was greater than 90%, and serum standard bicarbonate was 26 mmol/L. The fractional exhaled nitric oxide was 25 ppb. The blood eosinophil count was 200 cells/μl and the IgE was 164 IU [0-200], with elevated serum specific IgE to *Aspergillus fumigatus*, and non-reactive results to other allergens including grasses and dust mite. The haemoglobin level was 145 g/l. Computed tomography pulmonary angiogram (CTPA) did not detect pulmonary emboli, and the lungs were noted to be hyperinflated, but without marked emphysematous changes.

**Fig. 1:**
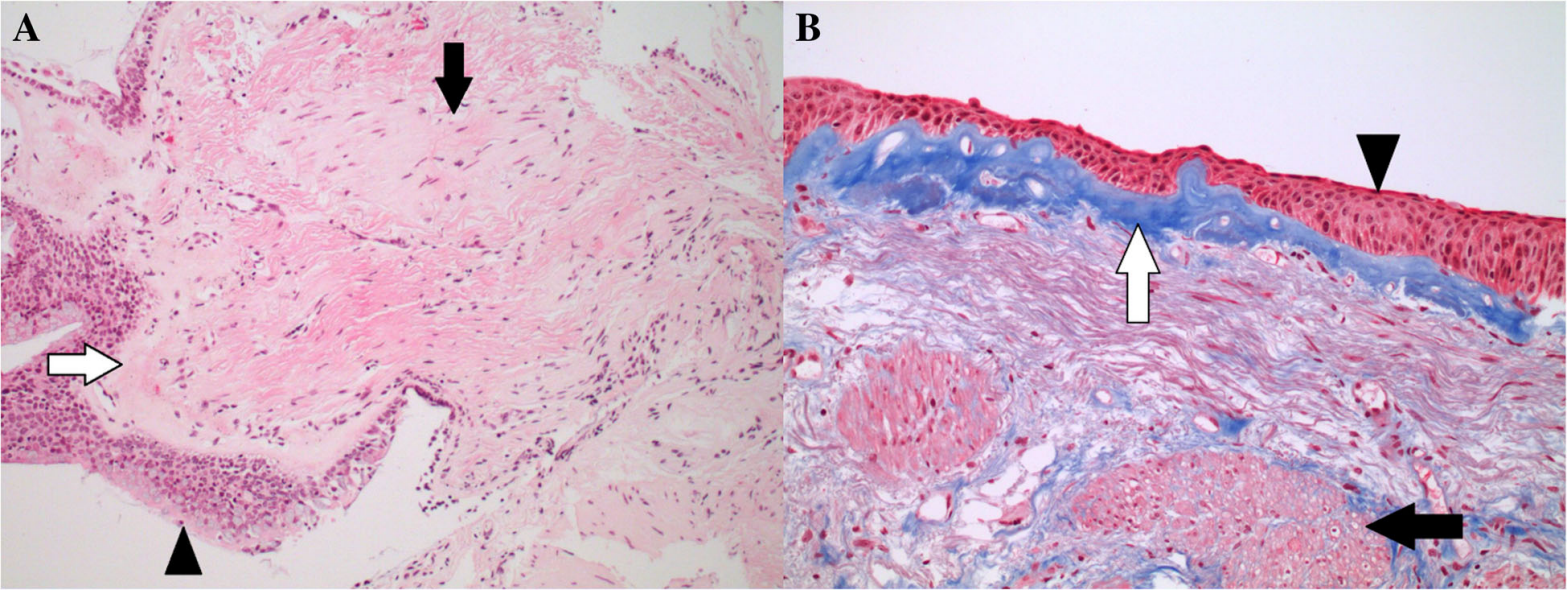
caption **a**: section of the endobronchial biopsy showing bronchial epithelial lining (black arrow head) with underlying marked subepithelial basement membrane thickening (white arrow) and smooth muscle hypertrophy (black arrow). Haematoxylin and eosin (H&E) stain, 200x magnification. **b**: Trichrome stain of the endobronchial biopsy highlighting the thickened subepithelial basement membrane (blue dye, white arrow). Also shown is squamous metaplasia of the bronchial epithelial lining (black arrow head) and underlying smooth muscle bundles (black arrow). 200x magnification

The patient was referred for pulmonary rehabilitation, which led to modest improvement, though she remained limited by exertional dyspnoea. At re-evaluation, consideration was given to whether the patient could benefit from an interventional approach such as endobronchial lung volume reduction surgery. A Quantitative CT for emphysema distribution and fissure integrity was requested. This demonstrated a relatively small lung fraction with <− 950 Hounsfield units (9.35%), although more prominent changes were observed in the left lower lobe. As this result did not unequivocally support a diagnosis of COPD, we undertook flexible bronchoscopy to obtain endobronchial biopsies from the left lower lobe. The 2.8 mm channel bronchoscope, Olympus BF-ITH190 (Olympus Australia, Victoria, Australia) was used with the 2.3 mm forceps, and four biopsies up to 5 mm in size were obtained from the subsegmental carina at LB8/9. Histopathology revealed very marked smooth muscle hypertrophy (Fig. 1) and significant thickening of the basement membrane typical of asthma. Additionally, squamous metaplasia due to cigarette smoking was evident.

As a direct result of the endobronchial biopsy, the treatment approach shifted to advanced therapies for severe asthma. The patient did not meet Australian funding criteria for anti-IgE or anti-interleukin 5 monoclonal antibodies, but she was however a suitable candidate for BT. The patient was treated over three sessions without complication and in keeping with the standard technique [1].

Six months after BT, the Asthma Control Questionnaire score had reduced from a baseline value of 3.0 to 1.6, where a score of 1.5 indicates well controlled asthma, and a change of 0.5 is regarded as clinically significant. The daily salbutamol use had decreased substantively to 0.5puffs/day. There had been no instances of exacerbations requiring antibiotics or corticosteroids. The prebronchodilator FEV_1_ improved slightly from 47 to 52% predicted and the RV improved markedly from 189 to 152% predicted.

## Discussion and conclusion

Asthma and COPD are very common diseases in our community and, when severe, pose significant management challenges for both the patient and the clinician. Fortunately, the range of therapies available to the patient with severe disease has substantially increased over the last decade with the arrival of monoclonal antibodies and of new interventional procedures. Each of these treatments, however, require an accurate diagnosis and the right phenotype. Whilst there is little overlap between the treatments for the two diseases at the severe end of each spectrum, there can be a great deal of overlap in the clinical manifestations of the two conditions.

Here we present a patient whose course of treatment was dramatically altered by the endobronchial biopsy revealing a histological diagnosis of asthma in contrast to a previous clinical diagnosis of COPD. It highlights the importance of accurate phenotyping in airways disease to guide treatment selection, but at the same time it is a reminder that the quest can be difficult.

Airway histology obtained with endobronchial biopsy is not routinely employed outside of research studies, although the technique is well within the remit of every practicing bronchoscopist. The concept is certainly not novel, having been demonstrated in 1960 to be of value in differentiating between asthma and chronic bronchitis, where asthmatics had numerous eosinophils in the lamina propria [2]. It is now generally accepted that features indicative of asthma include increased airway smooth muscle mass and thickened reticular basement membrane [3, 4]. Applying this to clinical practice has however produced mixed results. Bronchoscopic evaluation was used in one study to further evaluate refractory asthma phenotypes, leading to successful selection of patients for treatment with omalizumab, associated with significant improvement in asthma control [5]. Another study however noted considerable inter-observer variability in histological interpretation and definitions for asthma and COPD [6]. Overall, there is a paucity of large-scaled clinical trials in this area.

The small size of the endobronchial biopsy has contributed to interpretive difficulty in the past, but this predated the widespread availability of the wide channel bronchoscope and larger forceps, which enabled the substantive biopsies obtained in this case. An alternative method of obtaining larger diagnostic specimens can now also be found in mucosal cryobiopsies [7].

Delivering individualised therapy is the new benchmark in asthma treatment, and an increasing number of biomarkers are already part of the compendium of diagnostic tools used in clinical practice. We believe there is an additive and promising role in regular clinical practice for using airway histology to further refine airways disease phenotype when non-invasive assessment is confusing, particularly in the challenging domain of asthma-COPD overlap.

## Abbreviations

BT: Bronchial thermoplasty
COPD: Chronic obstructive pulmonary disease
CT: Computed tomography
DLCO: Diffusing capacity for carbon monoxide
FER: Forced expiratory ratio
FEV_1_: Forced expiratory volume in 1 s
RV: Residual volume
